# Clinical profile and outcomes of management of orbital cellulitis in Upper Egypt

**DOI:** 10.1186/s12348-017-0126-3

**Published:** 2017-03-09

**Authors:** Ahmed Mohamed Kamal Elshafei, Mohamed Farouk Sayed, Raafat Mohyeldeen Abdelrahman Abdallah

**Affiliations:** Department of Ophthalmology, Minia University Hospital, Minia City, Minia Governorate Egypt

**Keywords:** Orbital cellulitis, Subperiosteal abscess, Sinusitis

## Abstract

**Background:**

The purpose of this paper is to study the etiology, clinical findings, and outcomes of management of cases of orbital cellulitis treated in Minia University Hospital in Upper Egypt over the period of 6 years from July 2009 to July 2015. One-hundred two patients diagnosed to have orbital cellulitis were admitted to the hospital and treated on inpatient basis from July 2009 to July 2015. All patients were subjected to full ophthalmological examination, systemic evaluation, and ear, nose, and throat (ENT) consultation. Axial and coronal CT scan and orbital echography were done for all patients. All patients received medical treatments, and 20 patients needed surgical intervention.

**Results:**

The source of infection was paranasal sinusitis in 66 patients, trauma in 14 cases, panophthalmitis in 6 patients, and dental infection in 2 cases, and no definite source was detected in 14 cases. Subperiosteal abscess (SPA) developed in 16 patients. The final best corrected visual acuity improved in 58% of the cases, decreased in 4%, and remained unchanged in 38% of cases. No intracranial complication was recorded.

**Conclusions:**

Good presenting visual acuity and appropriate medical treatment together with early surgical intervention in cases of SPA are important factors to achieve favorable outcomes in orbital cellulitis. All cases with SPA had paranasal sinusitis, and contrary to previous studies, superior SPA location was the most common followed by the medial location.

## Background

Orbital cellulitis is a serious and sight-threatening condition caused by invasive infection of the postseptal tissues of the orbit [1]. The most common predisposing factor for orbital cellulitis is paranasal sinus disease. Infection may also arise from eyelids, face, retained orbital foreign bodies, or hematogenous spread from distant sources. Less common causes include dacryocystitis, dental infection, panophthalmitis, infected tumor, and mucormycosis [2, 3]. The most common causative organisms include *Streptococcus pneumoniae*, *Moraxella catarrhalis*, *Haemophilus* species, *Staphylococcus aureus*, group A *streptococcus*, and upper respiratory tract anaerobes [4]. Orbital cellulitis has been associated with serious complications including visual loss, cavernous sinus thrombosis, meningitis, frontal abscess, osteomyelitis, and even death [2, 3]. The mechanisms of visual loss may involve optic neuritis as a reaction to adjacent or nearby infection, ischemia resulting from thrombophlebitis along the valveless orbital veins, or compressive ischemia possibly resulting in central retinal artery occlusion [5]. Subperiosteal abscess (SPA) may result from the accumulation of pus between the orbital bones and periorbita [6]. Cavernous sinus thrombosis represents the most severe complication of orbital cellulitis and is suspected by bilateral involvement with ophthalmoplegia and loss of vision [7]. Broad-spectrum intravenous (IV) antibiotics that cover most gram-positive and gram-negative bacteria should be started once the diagnosis of orbital cellulitis is suspected. Surgical treatment is indicated for significant underlying sinus disease or SPA [8].

## Results

One-hundred two patients were included, 66 males (64.7%) and 36 females (35.3%). The mean age was 25.56 years + 18.87 (range 2 to 70 years). There were 52 adults and 50 children. All of them had unilateral orbital affection.

**Fig. 1:**
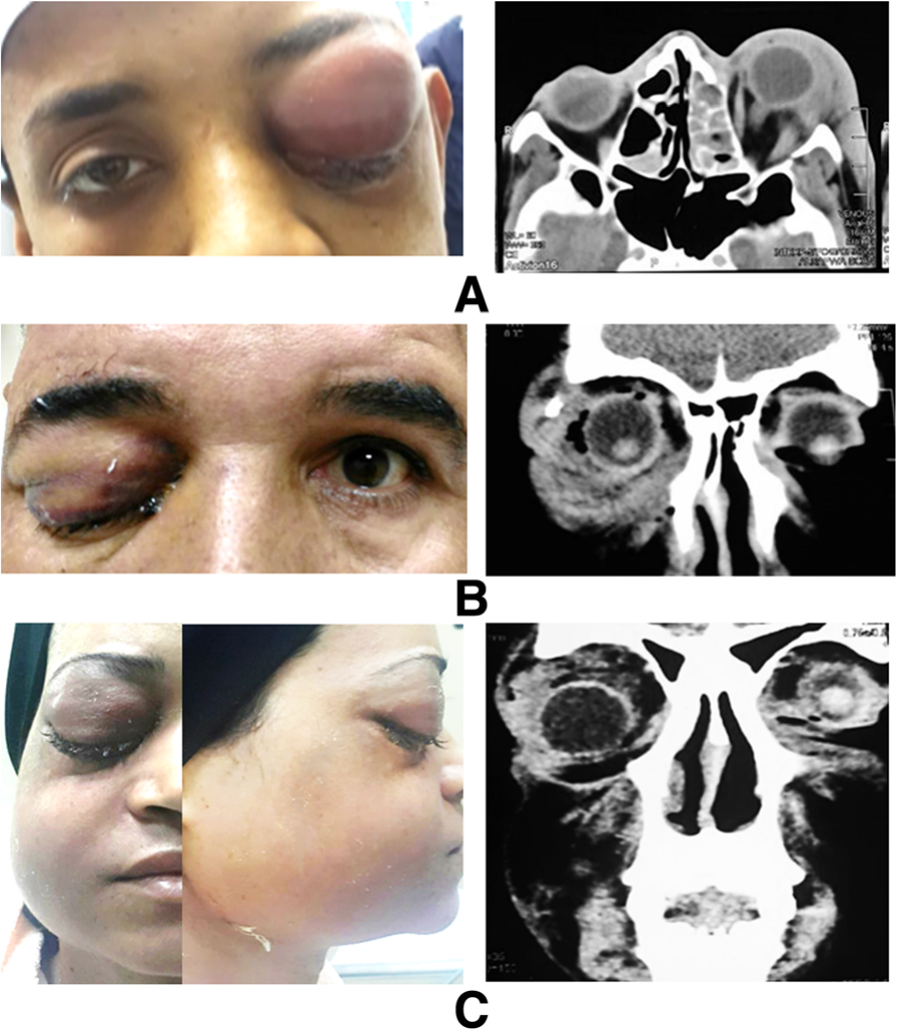
Etiology of orbital cellulitis. **a** Sinusitis. **b** Traumatic. **c** Dental infection

As regards the etiology of orbital cellulitis, 66 patients (64.7%) had radiological evidence of paranasal sinusitis (Fig. 1a), 36 patients had ethmoidal sinusitis, 6 patients had frontal sinusitis, and 24 patients had multiple sinuses affection, two of them had fungal orbital cellulitis (mucormycosis). Orbital trauma was the cause in 14 patients (13.7%), with 12 patients having penetrating trauma, two of them had a retained wood intraorbital foreign body while orbital cellulitis in 2 patients was secondary to blunt trauma with surgical emphysema of the orbit (Fig. 1b). In six patients (5.9%), orbital cellulitis was a complication of panophthalmitis secondary to extension of infection from the globe. Two patients (2%) developed orbital cellulitis due to dental infection in the form of dental abscess in one patient and complicated dental surgical procedure in the other (Fig. 1c). No definite source of infection was identified in 14 patients (13.7%), and the possibility of hematogenous spread from distant source was proposed.

**Fig. 2:**
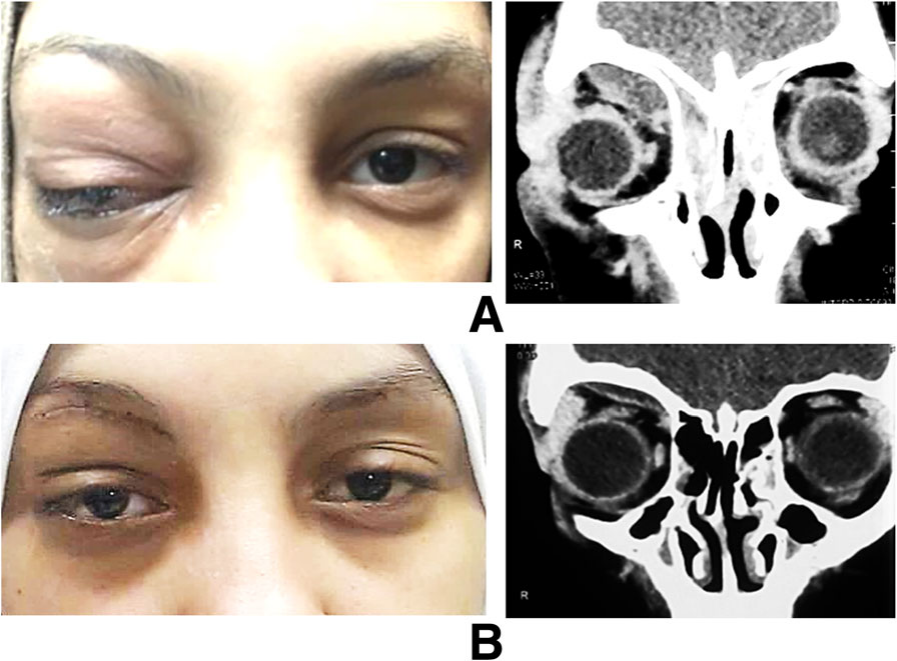
Subperiosteal abscess. **a** Preoperative. **b** Postoperative

Clinically, all patients presented with proptosis, periorbital edema, and tenderness with restriction of ocular motility. Table 1 summarizes the clinical data at the time of presentation. SPA developed in 16 patients, superior SPA in 10 patients, medial in 4 patients, and combined superior and medial abscesses in 2 patients (Fig. 2). All cases with SPA had paranasal sinusitis. Fourteen patients required surgical drainage, and two children with medial SPA responded to medical treatment.

**Table 1:** Summary of the clinical data

Clinical presentation	Descriptive statistics (Number = 102)
Sinusitis	
No	36 (35.3%)
Ethmoidal	36 (35.3%)
Frontal	6 (5.9%)
Mixed	24 (23.5%)
Fever	
No	40 (39.2%)
Yes	62 (60.8%)
Punctate keratitis	
No	80 (78.4%)
Yes	22 (21.6%)
Relative afferent pupillary defect	
No	84 (82.4%)
Yes	18 (17.6%)
Subperiosteal abscess	
No	86 (84.3%)
Upper	10 (9.8%)
Nasal	4 (3.9%)
Upper and nasal	2 (2%)

**Fig. 3:**
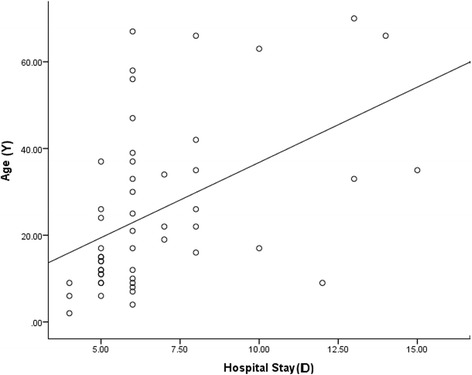
Correlation between the duration of hospital admission in days (*D*) and age of the patients in years (*Y*)

All patients received medical treatment, and 20 patients were subjected to surgical interference. Fourteen patients had surgical drainage of SPA and four patients required evisceration. Evisceration indicated in patients presenting with complete loss of vision (no perception of light) with corneal abscess and no response to medical treatment for more than 7 days. Orbital exenteration was done in two cases of mucormycosis. Histopathology confirmed the clinical diagnosis. The duration of hospital admission ranged from 4 to 15 days (mean 6.76 days ± 2.58). A clinically significant positive correlation was found between the duration of hospital admission and both of the age of the patient and surgical interference (Fig. 3 and Table 2).

**Table 2:** Correlation between the duration of hospital admission and age and surgical interference

Variable	Duration of hospital admission	
	*r*	*P* value
Age^a^	0.476	<0.001*
Surgical interference^b^	0.360	0.009*

^a^Pearson’s correlation

^b^Spearman’s rho correlation

^*^Significant correlation at *P* value ≤0.05

The presenting BCVA ranged from 6/6 to no perception of light (No PL). Table 3 shows the BCVA at first presentation and 2 weeks after discharge from hospital in 96 patients. Six patients were too young to numerically evaluate BCVA. Table 4 shows a comparison between the presenting and the final BCVA.

**Table 3:** Best corrected visual acuity (BCVA) at the first presentation and at the end of treatment

BCVA	Presenting	Final	*P* value
	BCVA	BCVA	
	(Number = 96)	(Number = 96)	
No PL	12 (12.5%)	14 (14.6%)	<0.001*
≤1/60	2 (2.1%)	0 (0%)	
≤6/60	4 (4.2%)	0 (0%)	
6/36	2 (2.1%)	0 (0%)	
6/24	4 (4.2%)	0 (0%)	
6/18	8 (8.3%)	0 (0%)	
6/12	26 (27.1%)	8 (8.3%)	
6/9	26 (27.1%)	32 (33.3%)	
6/6	12 (12.5%)	42 (43.8%)	

*BCVA* best corrected visual acuity, *No PL* no perception of light

^*^Significant difference at *P* value ≤0.05

**Table 4:** The overall visual outcome at the end of treatment

Visual outcome	Ranks	Number
Final BCVA versus presenting BCVA	Negative ranks	4^a^
	Positive ranks	56^b^
	Ties	36^c^
	Total	96

*BCVA* best corrected visual acuity

^a^Final BCVA < presenting BCVA

^b^Final BCVA > presenting BCVA

^c^Final BCVA = presenting BCVA

There was a clinically significant negative correlation between the final BCVA and the age of the patient, RAPD, SPA, surgical interference, and the duration of hospital admission (Table 5).

**Table 5:** Correlation between the final best corrected visual acuity and other clinical variables

Clinical variables	Final best corrected visual acuity	
	*r* ^a^	*P* value
SPA	−0.437	0.002*
Age	−0.353	0.014*
Hospital stay	−0.519	<0.001*
RAPD	−0.558	<0.001*
Surgical interference	−0.491	<0.001*
Corneal affection	−0.605	<0.001*
Fever	0.300	0.038*
Sinusitis	0.290	0.046*
Mucormycosis	−0.290	0.046*
Dental affection	−0.314	0.018*
Distant infection	−0.431	0.002*
Panophthalmitis	−0.216	0.140
Penetration eye trauma	−0.080	0.587
Trauma + foreign body	−0.056	0.704

*SPA* subperiosteal abscess, *RAPD* relative afferent pupillary defect

^a^Spearman’s rho correlation

^*^Significant correlation at *P* value ≤0.05

Throughout this study, no cases of cavernous sinus thrombosis or intracranial infection were present. Apart from patients subjected to orbital exenteration, all patients had disappearance of active inflammatory signs with recovery of proptosis and ocular motility restriction 2 weeks after the time of hospital discharge.

## Discussion

This study examined the clinical presentation and outcomes of management of cases of orbital cellulitis presented to Minia University Hospital over a period of 6 years. Minia University Hospital provides medical emergency and tertiary service for a population of five million people from nine towns in Upper Egypt.

In this study, paranasal sinusitis was present in 64.7% of patients of which ethmoidal sinusitis was present in 35.3%, frontal sinusitis in 5.8%, and pansinusitis in 23.5% of patients. Reported rates of an associated sinusitis with orbital cellulitis varied from 15 to 100% [9–13]. In the current study, orbital trauma was present in 13.7% of patients. Trauma was the predisposing factor in 19.7% of patients in the study of Chaudhry et al. and 21.7% of patients in the study of Hodges and Tabbara [3, 9]. Both studies were in Saudi Arabia. Trauma was present in 24% of patients of Pandian et al. in South India [12]. With the improvement in management of endophthalmitis, fewer cases present with orbital cellulitis secondary to endophthalmitis and panophthalmitis. These cases represent 6% in this study, compared to 17% in older studies [9].

Clinically, all patients presented with unilateral proptosis, periorbital edema and tenderness, and restriction of ocular motility. Fever was absent in 39.2% of patients. This may be due to the prescription of anti-inflammatory and antipyretic drugs before presenting to the hospital. SPA developed in 15.68% of patients. The incidence of SPA varies considerably in different studies; no case of SPA was reported in the study of Pandian et al. in India (71 patients), in the reviews from Children’s Memorial Hospital in Chicago (87 patients) and Children’s Hospital in Pittsburgh (104 patients) [12, 14, 15]. However, CT scan was done in only 20 patients in the first study; therefore, SPA could be easily missed. Fanella et al. studied the epidemiology and clinical data of pediatric orbital cellulitis in Manitoba. They identified 38 patients with orbital cellulitis. SPA occurred in 31.5% of patients [11]. According to Lee and Yen, frequent locations for the development of SPA include the medial orbital wall and the orbital floor due to the thin medial wall adjacent to the ethmoid sinus and the thin orbital floor above the maxillary sinus, respectively [16]. However, in the present study, superior SPA occurred in 62.5%, medial in 25%, and combined superior and medial abscesses in 12.5% of patients with SPA. No abscess related to orbital floor was detected. Superior SPA was present in four cases with isolated ethmoidal sinusitis with no frontal sinus affection.

The higher frequency of superior SPA in this study, in spite of the more frequent ethmoidal sinusitis, could be explained by the relatively easier separation of the periorbita from the orbital roof. The periorbita is firmly attached at the suture lines, the foramina, and the arcus marginalis. Elsewhere, it is loosely adherent and may be easily separated from the bone. The orbital aspect of the roof is generally smooth and presents only one suture between the frontal and sphenoid bone and this suture becomes obliterated with age. On the other hand, the medial wall is formed from parts of four bones with vertical sutures between them. Moreover, the posterior and anterior ethmoidal foramina which can permit transfer of infection from ethmoidal sinuses to the orbit open above the frontoethmoid suture being more related to the orbital roof [17].

Hospital admission and IV antibiotics are essential in management of orbital cellulitis. Many antibiotic combinations were used in different studies. Pandian et al. used a combination of IV penicillin and gentamicin in all patients. They used cephalosporins, vancomycin, and other antibiotics based on the sensitivity pattern or absence of clinical improvement with initial treatment [12].

Fanella et al. used different combinations of IV cefuroxime, clindamycin, cephalosporin, cloxacillin, cefotaxime, vancomycin, metronidazole, penicillin, and ampicillin. They changed initial antibiotics in 32% of cases based on infectious diseases consultations [11].

According to Lee and Yen, local trends in antimicrobial susceptibility are critically important considerations in orbital cellulitis treatment as different communities have distinctive flora and varied resistance profiles [16].

In this study, a combination of intravenous vancomycin and ceftazidime was used. Vancomycin covers *S. pneumoniae* and other *streptococci*, methicillin-resistant *S. aureus*, *Haemophilus influenzae*, and non-spore-forming anaerobes while ceftazidime covers *Pseudomonas* spp.; *H. influenzae*, *Klebsiella* spp.; *Enterobacter* spp.; *Proteus*; *Escherichia coli*; *Serratia* spp.; *Citrobacter* spp.; *S. pneumoniae*; methicillin-susceptible *S. aureus*; and *Streptococcus pyogenes* (group A beta-hemolytic *streptococci*). Antibiotic modification was done in cases with lack of clinical improvement or according to culture sensitivity in surgically treated patients.

The use of corticosteroids in the treatment of orbital cellulitis is controversial. The possibility of suppression of the immune system and worsening of the disease process should be considered. However, when used under the coverage of appropriate antibiotics, the use of systemic steroids decrease the inflammatory response and does not appear to adversely affect the outcome [18]. In this study, systemic steroids were used in cases with severe edema, marked drop of vision, or RAPD.

Once SPA develops, rapidly progressive visual and intracranial complications may occur. Therefore, the prompt surgical drainage when an abscess is first diagnosed by CT scan should be considered. Medical treatment alone may be sufficient in younger patients [6, 19–21]. Medial or inferior SPA is more likely to respond to medical therapy, while superior SPA requires surgical drainage [22]. In this study, 87.5% of patients with SPA required surgical drainage and only two children (12.5%) with small medial SPA were treated medically.

The duration of hospital admission was longer in patients subjected to surgical intervention and older patients who had less response to treatment.

The final visual outcome of the patients in this study revealed visual improvement in 58% and visual deterioration in 4% of cases. Thirty-eight percent of patients had unchanged BCVA. Factors adversely affected the final BCVA including the patient age, RAPD, and SPA. Six patients presented late with complete visual loss in the affected eye (No PL) and had no visual improvement at the end of treatment, and only one patient with SPA lost his vision due to optic atrophy after late presentation and delayed surgical interference.

Pandian et al. in South India reported improved visual outcome in 60% of 33 patients with orbital cellulitis. The causes for poor outcomes in their study were panophthalmitis (two cases), perforated scleral abscess (one case), phthisis bulbi (two cases), choroiditis (two cases), orbital abscess (four cases), and retinal detachment (one case). The first three patients were treated with evisceration. Six patients lost vision due to these complications [12]. Ferguson and McNab studied treatment outcomes in 52 patients with orbital cellulitis in Melbourne. They had only one patient who lost his vision due to enucleation for endophthalmitis that had caused orbital cellulitis and one patient who developed meningitis [2]. Hartstein et al*.* reported three patients with SPA who developed intracranial abscess [23]. No intracranial involvement was detected in the current study.

## Conclusion

Favorable outcomes in orbital cellulitis are associated with good presenting visual acuity and appropriate medical treatment together with early surgical intervention in cases of SPA. All cases with SPA had paranasal sinusitis, and contrary to previous studies, superior SPA location was the most common followed by the medial location.

## Methods

The study included 102 patients diagnosed to have orbital cellulitis and were treated in the Ophthalmology Department of Minia University Hospital from July 2009 to July 2015.

All patients were admitted to the hospital and treated on inpatient basis. All patients were subjected to ophthalmological evaluation, systemic evaluation, and ear, nose, and throat (ENT) consultation. Ophthalmological evaluation included history taking, orbital, and ocular examination. Orbital examination included inspection, palpation, and exophthalmometry. Ocular examination included slitlamp examination, pupillary reaction testing, ocular motility assessment, best corrected visual acuity (BCVA) recording, intraocular pressure measurement, and fundus examination. Axial and coronal orbital CT scans and orbital echography were done for all patients. All patients received medical treatments, and 20 patients needed surgical intervention.

### Treatment protocol

All patients received parenteral broad-spectrum antibiotics covering gram-positive, gram-negative, and anaerobic bacteria in the form of a combination of IV vancomycin and ceftazidime. IV clindamycin was added in 12 patients and oral metronidazole in 8 patients according to the response to treatment or culture results in the surgically treated cases. Oral antibiotic (combination of amoxicillin and clavulanic acid) was given to all patients for 2 weeks after discharge from hospital. Topical antibiotic ointment, tear substitutes, and patching were considered in cases with corneal exposure. Topical and/or systemic ocular hypotensive drugs were given if there is associated rise of the intraocular pressure. Systemic steroids were added in cases with severe edema, cases presented with drop in the BCVA (≤6/18), and patients with relative afferent pupillary defect (RAPD, 42 patients). IV amphotericin B was given to two cases of fungal orbital cellulitis (mucormycosis).

Surgical intervention was performed in 14 patients with SPA in the form of surgical drainage under general anesthesia through extraperiosteal approach. Evisceration was done in four cases presented with panophthalmitis and complete loss of vision (no perception of light) with no response to medical treatment. Orbital exenteration with surgical debridement of the necrotic tissues was done in two cases of mucormycosis in collaboration with ENT and maxillofacial surgeons.

This study was approved by the local ethics committee of Minia University and was adhered to the tenets of the Declaration of Helsinki. Informed consent was obtained from each patient after explanation of the nature and possible consequences of the study.

#### Statistical method

The patient demographics, etiology, the visual acuity, the frequency of the development of SPA, the duration of hospital admission, the rate, and outcomes of surgical intervention were statistically analyzed using SPSS program (Statistical Package for Social Sciences) software version 20. Analyses were done for qualitative variables at different times using the Wilcoxon signed-rank test. Correlation between two quantitative variables was done using Pearson’s correlation coefficient, while correlation between qualitative ordinal variables was done using non-parametric Spearman’s rho correlation coefficient.
